# Neuroimaging in Functional Movement Disorders

**DOI:** 10.1007/s11910-019-0926-y

**Published:** 2019-02-12

**Authors:** Jacob J. Roelofs, Tiago Teodoro, Mark J. Edwards

**Affiliations:** 1 Southmead Hospital, North Bristol NHS Trust, Bristol, UK; 2 Neuroscience Research Centre, Institute of Molecular and Clinical Sciences, St George’s University of London;, Cranmer Terrace, London, SW17 0QT UK; 3 Instituto de Medicina Molecular, Faculdade de Medicina de Lisboa, Universidade de Lisboa, Lisbon, Portugal; 4 Atkinson Morley Regional Neuroscience Centre, St George’s University Hospitals, London, UK

**Keywords:** Functional movement disorders, Conversion disorder, Psychogenic, MRI, fMRI, Imaging

## Abstract

**Purpose of Review:**

Functional movement disorders are common and disabling causes of abnormal movement control. Here, we review the current state of the evidence on the use of neuroimaging in Functional movement disorders, particularly its role in helping to unravel the pathophysiology of this enigmatic condition.

**Recent Findings:**

In recent years, there has been a shift in thinking about functional movement disorder, away from a focus on high-level psychological precipitants as in Freudian conversion theories, or even an implicit belief they are ‘put-on’ for secondary gain. New research has emphasised novel neurobiological models incorporating emotional processing, self-representation and agency.

**Summary:**

Neuroimaging has provided new insights into functional movement disorders, supporting emerging neurobiological theories implicating dysfunctional emotional processing, self-image and sense of agency. Recent studies have also found subtle structural brain changes in patients with functional disorders, arguing against a strict functional/structural dichotomy.

## Introduction

Functional neurological disorders (FND) are common, accounting for 16% of all referrals to general neurology clinics but have historically been rather neglected in research and clinical service development [1]. Research and clinical work with people with functional movement disorders (FMDs) has helped to lead a shift in thinking about FND by emphasising positive diagnostic criteria, and by development of neurobiological-level theories of the pathophysiology of FND. Neuroimaging has formed a key part of this work, providing data that has informed pathophysiological understanding.

A wide variety of terms have been used to describe FNDs including ‘hysterical’, ‘conversion’ and ‘somatisation’, each with implications about the underlying mechanism. ‘Functional neurological symptoms’ is now used as a neutral umbrella term for all ‘non-organic’ symptoms which is non-judgemental and acceptable to patients. This has helped to create a shift in thinking about FNDs, away from a focus on high-level psychological precipitants as in Freudian conversion theories, or even an implicit belief they are ‘put-on’ for secondary gain. New research has emphasised novel neurobiological models incorporating emotional processing, self-representation and agency [2]. Advances in neuroimaging have provided new insights into changes in brain activity, functional connectivity and brain structure in FND. The diagnosis of FND is based on identifying clinical signs which are inconsistent with organic disease, and which may vary over time or with attention [3]. As functional movement disorders (FMDs) can be assessed objectively with physical examination in a clinic setting, changes over time and the effects of attention can be clearly documented. This makes them an ideal model to investigate the underlying mechanism of FNDs in general.

## Using Neuroimaging to Exclude Co-Morbid Organic Disease

In all patients who present with a new neurological symptom, the first concern is to exclude conditions requiring urgent treatment. In FND, the clinical history and examination is often clear enough to do this. If there is any uncertainty, especially as organic and functional symptoms may co-exist, structural neuroimaging such as CT and MRI can be used to exclude a co-morbid organic disorder in specific circumstances.

This is particularly important where multiple disease processes produce a mixed clinical picture, or where an underlying organic process is associated functional ‘overlay’. Functional symptoms may be triggered by a ‘stressor’ which can be physical, such as limb injury, psychological or both [4]. Elmalı and colleagues recorded the case of a patient with a functional tremor, but imaging abnormalities prompted further investigation, which revealed the presence of Wilson’s disease [5]. Kwon and colleagues used structural imaging to diagnose a rare subtype of motor neurone disease in a patient with a functional tremor [6]. In both these cases, the implication is not that the original diagnosis of a functional movement disorder was incorrect, but that functional symptoms can co-occur with other illnesses, some of which may be treatable in their own right.

In certain patients, specific imaging techniques can provide greater diagnostic certainty—an example is idiopathic Parkinson’s disease and functional parkinsonism. Functional overlay appears to be commoner than expected in patients with idiopathic Parkinson’s disease while pure functional parkinsonism seems comparatively rare [7, 8]. The fact that idiopathic Parkinson’s disease has effective treatment options makes the distinction important.

Dopamine transporter-single-photon emission computer tomography (DaTSCAN) uses a radioactive ligand which binds to dopamine transporters and is highly sensitive to the pattern of nigrostriatal denervation characteristic of idiopathic Parkinson’s disease [9, 10]. DaTSCANs are normal in functional or drug-induced parkinsonism, making it a highly effective tool in certain cases [11, 12]. Other techniques such as [123I]-FP-CIT SPECT, which also uses a radiolabelled ligand, can add further accuracy, especially between idiopathic and drug-induced parkinsonism [13]. Umeh and colleagues investigated three patients with a mixed clinical picture of functional and neurodegenerative parkinsonism. Two patients had abnormal DaTSCANs and were diagnosed with idiopathic Parkinson’s disease with co-morbid functional symptoms, while the third patient had normal imaging leading to a diagnosis of functional parkinsonism [14]. Gaig and colleagues used [123I]-Ioflupane SPECT imaging in nine patients with suspected functional parkinsonism. The results were normal in 8 patients, but one had bilaterally decreased tracer uptake, and further investigation led to the identification of a *parkin* gene mutation [15]. These studies show how targeted imaging can aid diagnosis, even in complex or overlap cases.

## Functional Imaging and Novel Neurobiological Models of FMDs

Recent neuroimaging studies have implicated abnormal emotional processing, sense of agency, and top-down regulation from frontal areas as important pathophysiological components in FMD [16]. Functional imaging techniques such as fMRI and PET allow abnormalities in regional brain activation, and functional connectivity between brain areas, to be measured. In the future, these differences could potentially be used to aid the diagnosis of FMDs. Wergrzyk and colleagues used resting state fMRI to distinguish FMD from matched controls with over 68% sensitivity and specificity [17]. This was primarily using connectivity around the right caudate, amygdala, prefrontal and sensorimotor regions which were hyperconnected in FMD [17].

### Abnormal Emotional Processing

There has been considerable interest in how traumatic experiences and emotional factors might be involved in the pathogenesis of FMD. Voon and colleagues found that patients with functional weakness showed reduced activity in the left supplementary motor area (SMA), which is involved in movement initiation, when performing movement tasks [16]. They also had increased activity in the right amygdala, insula and bilateral posterior cingulate cortices, which are involved in emotional processing. There was reduced functional connectivity between the left SMA and the bilateral dorsolateral prefrontal cortices when performing internally generated compared to externally generated movements. The authors proposed that in ‘emotionally arousing’ contexts, abnormal learned motor patterns interfere with normal movement initiation in the SMA, which is hypoactive and has reduced connection to the frontal lobe [16].

Increased activity in the amygdala, insula and cingulate cortices may indicate FMD patients have greater involvement of emotional processing areas in movement. In 1997, Marshall and colleagues recorded regional cerebral blood flow (rCBF) of a patient with unilateral functional leg weakness [18]. She had normal activation while moving her good leg and while preparing to move her weak leg, but when attempting to move her weak leg there was no activation of the primary motor cortex. Instead, the orbitofrontal and anterior cingulate cortices activated, which are involved in emotional processing and particularly previous traumatic emotional experiences [18]. It was suggested these areas inhibited activation of the primary motor cortex, preventing movement [18].

A further study by Voon and colleagues in 2010 found that patients with functional weakness showed less difference in activation of the right amygdala on exposure to fearful or happy faces than healthy controls [19]. These patients also had a tendency towards greater activation of the right amygdala than controls, suggesting increased excitability and decreased habituation [19]. There was also greater functional connectivity between the right amygdala and the right SMA in functional patients than controls [19]. This could imply changes in emotional processing and greater emotional involvement in movement initiation.

In 2018, Espay and colleagues investigated emotional processing in patients with functional tremor, comparing them with patients with essential tremor and controls in finger tapping, basic-emotion and intense-emotion tasks [20•]. Functional patients showed increased activation of the cerebellum in the motor task compared to those with essential tremor [20•]. In the basic-emotion task, functional patients had increased activation of the paracingulate and left Heschl’s gyrus compared to controls, but decreased activation of the right precentral gyrus compared to essential tremor patients. Again, patients with functional symptoms showed changes in areas involved in emotional processing. The study was repeated using patients with functional and primary organic dystonia [21]. Patients with functional dystonia had altered activation during the basic and intense emotion tasks in several areas, including decreased activation of the left insular and motor cortices [21]. In 2018, LaFaver and colleagues published a study comparing fMRI of 9 FMD patients pre- and postrehabilitation [22••]. They found a significant shift in activation during a Go/No-Go task from the visual cortex, cerebellar vermis and hippocampus to caudate, putamen and SMA, indicating a shift from bottom-up to top-down control of motor function [22••]. Interestingly, they found that improved outcome was related to increased functional connectivity posttreatment between primary motor cortex and the amygdala [22••].

These studies support the link between emotional processing and FMDs by showing altered activation of brain areas involved in emotional processing and increased functional connectivity between emotional and movement-related brain areas. This could provide a mechanism for emotional dysregulation to interfere with movement.

### Abnormal Sense of Agency

Functional motor symptoms show some features of voluntary movement, such as distractibility and variability with attention. Despite this, patients perceive their movements, or lack of movement, as involuntary. This suggests that part of the pathology of FMDs may be related to a patient’s sense of agency. ‘Agency’ is the experience of being the cause of our own actions [23, 24] and while it is linked to intention, there is evidence it is a ‘post-intention’ process. The sense of agency is a process of retrospective assessment of the action and the expected and actual sensory consequences [25]. This depends on the comparison of actual sensory feedback with the predicted sensory signal, known as ‘efference copy’ [26, 27]. This process has been localised to the temporo-parietal junction (TPJ), prefrontal cortices and the cerebellum [28, 29].

Advances in functional imaging allow us to investigate this feature of FMD and identify the brain regions involved. One benefit is to show conclusive differences between the neuroimaging correlates of functional weakness and a subject intentionally feigning weakness.

Voon and colleagues found reduced activation of the right temporo-parietal junction (TPJ) and reduced connectivity between it and the sensorimotor cortex and cerebellum in patients with functional tremor compared to voluntarily mimicked tremor in the same patients [28]. Another study in 2016 compared the functional connectivity of the right TPJ at rest in FMD patients with controls [30]. It found decreased connectivity with bilateral sensorimotor areas, the cerebellum, the SMA and the right insula, supporting impaired sensorimotor feedback integration. Nahab and colleagues published a study comparing fMRI findings of FMD patients with controls during a virtual reality movement task designed to modulate sense of agency [31, 32]. FMD patients showed dorsolateral prefrontal cortex and presupplementary motor area dysfunction which was unaffected by the loss of movement control [32].

Baek and colleagues used the Libet’s Clock task to assess FND patients’ accuracy in measuring their agency over the movement of a dot on the screen [33]. Compared to healthy controls, they had reduced activity of the inferior parietal lobule of the right TPJ when comparing movement verses intention trials. They also found a reduction in rest-state connectivity between the right TPJ and the dorsolateral prefrontal and anterior cingulate cortices, with increased connectivity with the premotor cortex and SMA. Reduced activity in areas involved in integrating sensory feedback after movement in functional patients supports a role for dysfunctional sense of agency in these symptoms.

Van Beilen and colleagues reported decreased activity in the supramarginal gyrus in FND patients [34], thought to be involved in integration of somatic and environmental cues leading to impaired movement initiation. Activity in the precuneus, which is also linked to sense of agency, was reduced in functional patients and importantly, was increased in feigning controls [34]. Feigning controls also had increased activity in the presupplementary motor area, which has been linked to motor intention [25, 34]. Hassa and colleagues in 2016 investigated motor inhibition specifically—comparing patients with functional motor weakness with controls feigning weakness [35••]. Passive movement of the weak hand activated the inferior frontal gyrus, but in different areas in functional and feigning controls. They also observed increased activity in the medial prefrontal cortex in the functional patients, which may also represent dysfunctional sense of agency [35••].

Another study compared patients with functional tremor, essential tremor and controls during a tremor-inducing motor task and at rest [36]. During the motor task, patients with functional tremor showed reduced rCBF in anterior regions of the default mode network, but at rest had increased rCBF in the inferior frontal gyrus and left insula. The authors related the differences to abnormalities in the default mode network in functional patients. The default mode network, also known as the task-negative network, is a set of brain regions noted to be active together when a person is wakeful but at rest. It is thought to be involved in thinking about the self, forward planning and theory of mind. This supports functional symptoms arising from abnormalities in self-representation and agency [36].

These results highlight the difference between patients feigning weakness and those with functional symptoms. They demonstrate differences in the activation and connectivity of a network, including the right TPJ and precuneus, which is believed to integrate sensory and motor feedback to generate a sense of agency.

### Abnormal Inhibitory Activity in Functional Weakness

Burgmer and colleagues analysed fMRI activity during observation and imitation tasks in patients with functional hand weakness [37]. They found reduced activation of cortical areas related to hand movement compared to controls when observing movement, but no evidence of frontal lobe movement inhibition [37]. This was felt to demonstrate impairment of movement conceptualisation rather than active inhibition from the frontal lobe. Cojen and colleagues compared fMRI activity on a Go/No-Go task in one patient with left-sided functional weakness with controls feigning weakness [38]. There was preserved right motor cortex preparatory activation, suggesting preserved intention. During inability to move the affected left hand in Go trials, there was increased activity of the precuneus and ventrolateral frontal gyrus, and increased functional connectivity between the posterior cingulate, precuneus and ventromedial prefrontal cortex [38]. During No-Go trials, there was normal inhibitory frontal activation in the right hand but none for the weak left hand. In contrast, for the feigning weakness controls, there were similar patterns of activity for No-Go and Go trials with the feigned weak hand. The authors argued this showed that functional weakness was not the direct result of frontal inhibition, as in the voluntary decision to not go, but due to activation of midline structures related to self-presentation, agency and emotional regulation.

Spence and colleagues used PET imaging to show that attempted movement of a functionally weak hand was associated with hypoactivation of the left dorsolateral prefrontal cortex, while attempted movement of a feigned weak hand was associated with hypoactivation of the right anterior prefrontal cortex [39]. They argued this linked motor inhibition in functional weakness to the prefrontal cortex [39].

### Role of Subcortical Structures in Functional Neurological Disorders

There has been comparatively little research looking at the involvement of subcortical structures in functional movement disorders. Vuilleumier and colleagues used SPECT scanning to investigate patients with functional unilateral sensorimotor impairment during hand vibratory stimulation and found decreased activation of the contralateral thalamus and basal ganglia [40]. When this was repeated after symptom improvement activation had normalised, suggesting the abnormality was related to the generation of symptoms, rather than a trait phenomenon [40].

Schrag and colleagues used PET to compare functional and organic dystonia patients at rest, during fixed posturing and paced movements [41]. Both forms of dystonia showed increased activation of the right dorsolateral prefrontal cortex during movement compared to controls, suggesting prefrontal cortex activation is non-specific. Patients with functional dystonia showed greater activation in the cerebellum and basal ganglia compared to patients with organic dystonia, who showed greater activation in the primary motor cortex.

## Structural Changes in Functional Movement Disorders

Considerable efforts have been made to promote the use of the word “functional” in recent times, but emerging imaging evidence suggests that replacing one dualistic simplification (physical vs. psychological) with another (functional vs. structural) is not without its problems. New studies have highlighted structural changes in the brains of patients with FMDs. Morphometric MRI techniques have detected subtle changes in volume and cortical thickness, particularly in areas thought to be involved in stress-related neuroplasticity [42, 43•, 44].

Both FMD and psychological conditions such as posttraumatic stress disorder (PTSD) have been associated with changes in areas responsible for emotional processing, such as the insula, cingulate cortex and amygdala [42]. These areas are believed to be subject to stress-mediated neuroplasticity, which may provide a mechanism for the changes in their activity and, over time, structure. Perez and colleagues measured the volume of the left insula cortex in women with FND. In one subgroup, there was a correlation of lower volumes with increasing symptom severity and childhood abuse burden [42]. In patients with PTSD symptom, severity was associated with lower volumes of the dorsal anterior cingulate cortex, and lifetime adverse event magnitude was inversely correlated with left hippocampal volumes [42]. Another study from Perez and colleagues used post hoc stratified analysis to show that physical impairment in FND patients was inversely correlated with left anterior insula volume compared to controls [43•]. Further, within group analysis of FND patients showed a correlation between mental health impairment, increased trait anxiety and increased right amygdala volumes [43•]. In a subsequent study published, Perez and colleagues found that FMD patients with high somatoform dissociation scores had reduced left anterior cingulate cortex thickness compared to controls [44]. They also reported a positive correlation between scores for depersonalisation/derealisation and right lateral occipital thickness [44]. Williams and colleagues showed that in FMD patients, changes in the hippocampal cortical thickness and volume were associated with differences in self-reported attachment styles [45].

Aybek and colleagues used MRI voxel based cortical thickness analysis (VBCT) to show significantly increased thickness in the premotor cortex of patients with functional hemiparesis compared to controls [46]. Another study by Nicholson found significant reduction in left thalamus volume in patients with FMD when compared to controls, which did not vary with laterality, severity of symptoms or handedness [47]. Sarasso and colleagues showed that compared to healthy controls, functional dystonia patients had reduced volume of the right thalamus and bilateral caudate with bilateral thinning of the precentral and frontoparietal cortices [48]. This is in addition to reduced functional connectivity between the right basal ganglia, dorsolateral prefrontal cortex and precuneus [48].

There is a growing body of evidence to suggest that subtle changes in brain area volume and cortical thickness may occur in functional neurological disorders. These may be secondary changes due to limb disuse in the case of thalamic or motor areas, but the changes in cingulo-insular structures more likely reflects stress-mediated neuroplasticity. This provides further evidence linking abnormal emotional processing to the neurobiology of FMDs.

## Conclusions

Functional neurological disorders are an exciting and rapidly expanding field of research within neuroscience. Functional movement disorders are especially amenable to investigation due to the nature of the symptoms and the comparative ease of constructing experimental paradigms, and this has placed them at the forefront of research. Neuroimaging has for a long time been a part of diagnosis of functional movement disorders primarily for assisting in the diagnosis of co-morbid organic disorders. More targeted forms of imaging, such as DaTSCANs are additionally useful in specific circumstances.

More recent developments in functional imaging have provided many new insights into the brain networks involved, as well as providing some supportive evidence for pathophysiological theories. Most recently, volumetric analysis has detected possible structural changes in the brains of FND patients.

Caution is needed, because as with all research in people with FMD, the patients themselves are very heterogenous in terms of symptoms and co-morbidities, with a high incidence of psychiatric co-morbidities and potentially relevant early life or more recent stressors. So far imaging research in FMD has used diverse paradigms, patient types and relatively small numbers for each study. It is also important that functional imaging can only give correlations, and these are vulnerable to confounding factors. Nevertheless, there is converging evidence about the underlying neurobiology of functional movement disorders and neuroimaging will continue to play a key role as the field develops.
